# Effect of new antioxidants: phenolipids on quality of fried French fries and rapeseed oil

**DOI:** 10.1007/s13197-020-04765-z

**Published:** 2020-09-01

**Authors:** Aleksandra Szydłowska-Czerniak, Dobrochna Rabiej

**Affiliations:** Faculty of Chemistry, Nicolaus Copernicus University in Toruń, 7 Gagarin Street, 87-100 Toruń, Poland

**Keywords:** Antioxidant activity, French fries, Rapeseed oil, Phenolipids, Oxidation parameters, Frying

## Abstract

The purpose of this work was to evaluate the effect of five novel synthetic antioxidants: octyl sinapate (OSA), octyl ferulate (OFA), octyl caffeate (OCA), cetyl sinapate (CSA) and cetyl ferulate (CFA) added to refined rapeseed oil for the first time on antioxidant activity (AA) and total phenolic content (TPC) in French fries. French fries after frying in enriched oils had higher the AA determined by three assays: 2,2′-azino-bis-3-ethylbenzothiazoline-6-sulfonic acid (ABTS = 2907–20,029 μmol TE/100 g), 2,2-diphenyl-1-picrylhydrazyl (DPPH = 657–6886 μmol TE/100 g), ferric reducing antioxidant power (FRAP = 332–2659 μmol TE/100 g) and total phenolic content (TPC = 44–378 mg SA/100 g) than those prepared in refined rapeseed oil (ABTS = 2146 μmol TE/100 g, DPPH = 403 μmol TE/100 g, FRAP = 218 μmol TE/100 g, TPC = 14 mg SA/100 g). Moreover, the presence of phenolipids in rapeseed oil decreased the oil content in fried French fries by 22–45%. However, the AA and TPC in the fortified oils significantly increased, whereas slower changes in oxidation parameters of supplemented oils after frying were observed. A new lipophilic antioxidants can be useful for the development of management system for the preparation of French fries and the prolongation of frying oil shelf life.

**Electronic supplementary material:**

The online version of this article (10.1007/s13197-020-04765-z) contains supplementary material, which is available to authorized users.

## Introduction

Frying is one of the most popular process of food preparation, wherein the food is cooked while floating or being immersed in hot oil. Unfortunately, high temperature and oxygen exposition during the frying resulting in oxidation, polymerization, degradation, hydrolysis, which produce a lot of nutritionally harmful compounds with negative impact on quality of oil and fried food.

A well-known synthetic (butylated hydroxytoluene, BHT, butylated hydroxyanisole, BHA, tertiary butyl hydroquinone, TBHQ) and various natural (tocopherols or other phenolic compounds) antioxidants are introduced to oils in order to retard their autoxidation and enhance the shelf life of fried products (Aydeniz and Yilmaz 2012). However, the addition of synthetic antioxidants is strictly regulated by governments due to the potential health risk of these compounds in foods. Therefore, the efficacy of natural antioxidants extracted from olive leaf, olive mill waste water, hazelnut leaf, hazelnut green leafy cover, wild rose hip, berries, fruits, herbs, spices, canola distillate, canola meal and rosemary for extension of shelf life of frying oils and fried products has been extensively studied (Aachary et al. 2014; Aladedunye et al. 2014; Aladedunye and Matthäus 2014; Aydeniz and Yilmaz 2012; Chiou et al. 2009; Matthäus et al. 2014; Redondo-Cuevas et al. 2017; Sordini et al. 2019). The supplementation of frying oils with natural antioxidants improved the quality of prepared food products due to the absorption of fats with active compounds during thermal treatment. French fries fried in rapeseed oils with purified native and lipophilized extracts from rowanberry and crabapple had higher content of tocopherols than those prepared in refined rapeseed oil without antioxidants (Aladedunye et al. 2015; Aladedunye and Matthäus 2016). Furthermore, French fries after frying in sunflower, palm and olive oils fortified with olive leaf extracts were richer source of polyphenols, tocopherols, phytosterols, squalene and exhibited higher antioxidant activity (AA) in comparison with control oil samples (Chiou et al. 2009).

However, the application of plants’ antioxidants, mainly phenolic acids in oil-based food products are limited due to their low solubility in hydrophobic media. Recently, lipophilization of phenolic acids, especially through esterification with fatty alcohols has been a widely recognized method to enhance their solubility in non-aqueous media and applicability in foods containing oils and fats (Alemán et al. 2015; Menezes et al. 2011; Sørensen et al. 2014; Szydłowska-Czerniak et al. 2018). Moreover, the addition of the lipophilized phenolic compounds (phenolipids) to mixture of rapeseed and linseed oils, mayonnaise and milk enriched with fish oil significantly affected the antioxidant properties of enriched fat products (Alemán et al. 2015; Sørensen et al. 2015; Szydłowska-Czerniak et al. 2018). The antioxidative effect of synthesized phenolipids depended on their chemical structure (mainly the alkyl chain length), concentrations and type of real samples (bulk oil or emulsion).

Although oxidative stability and amounts of tocopherols and phytosterols in the fortified rapeseed oils during frying have widely been investigated, to the best of our knowledge there are no reports on the estimation of the impact of phenolipids added to frying medium on the AA of fried French fries, enriched rapeseed oil and fat uptake.

Therefore, the purpose of the present work was to investigate the effect of five novel synthetic antioxidants: octyl sinapate (OSA), octyl ferulate (OFA), octyl caffeate (OCA), cetyl sinapate (CSA) and cetyl ferulate (CFA) added to rapeseed oil on the antioxidant properties of fried French fries and absorbed oil as well as oil uptake during frying. Moreover, the changes in antioxidant potential and oxidative status of rapeseed oils without and with phenolipids before and after frying were estimated. The AA of the studied samples were determined by 2,2′-azinobis-3-ethylbenzothiazoline-6-sulfonic acid (ABTS), 2,2-diphenyl-1-picrylhydrazyl (DPPH) and ferric reducing antioxidant power (FRAP) methods, while total phenolic content (TPC) was analyzed by Folin–Ciocalteu (FC) assay.

## Material and methods

### Reagents

All reagents of analytical or HPLC grade were purchased from Sigma-Aldrich (Poznań, Poland).

#### Chemical synthesis, purification and analysis of phenolipids

The novel lipophilic antioxidants: OSA, OFA, OCA, CSA and CFA were synthesized by Fischer esterification following the procedure described in our previous report with some modifications (Szydłowska-Czerniak et al. 2018). In brief, to sinapic acid—SA (6 mmoL), ferulic acid—FA (6 mmoL) and caffeic acid—CA (6 mmoL) with 1-octanol (48, 24 and 66 mmoL for SA, FA and CA, respectively) or 1-hexadecanol (66 and 33 mmoL for SA and FA, respectively) dried over 3 Å molecular sieves prior to reactions, in a two-neck flasks equipped with a magnetic stirring bar, reflux condenser and a thermometer, 25 µL of the sulphuric acid solution in alcohol (10 mol/L) was added during stirring. Moreover, activated molecular sieves (3 Å, 40 mg/mL) were introduced to the reaction mixtures to remove water formed during esterification. The reaction mixtures were stirred during the whole reaction time (3 h for SA and FA and 2 h for CA) and incubated at 100 °C. Reaction mixtures were cooled to room temperature, diluted with ethyl acetate (octyl esters) or tert*-*butyl methyl ether (cetyl esters), washed with brine and water (3 times) for removal of acids and dried over MgSO_4_. The solvents were evaporated under reduced pressure.

The synthesized octyl and cetyl esters (OSA, OFA, CSA and CFA) were purified by flash column chromatography on silica gel (pore size 60 Å, Kieselgel, Macherey–Nagel, Germany, column: 400 mm long × 20 mm in diameter, Witko, Poland) using as an eluent dichloromethane/ethyl acetate = 90:10, whereas OCA was purified by crystallization from heptane.

Qualitative analysis of the obtained phenolic acids esters was carried out by thin-layer chromatography (TLC) on pre-coated TLC-plates, silica gel 60 with fluorescent indicator UV254, trade name ALUGRAM R SIL G/UV_254_ (Macherey–Nagel, Germany), using a dichloromethane/ethyl acetate (90:10) as an eluent. The spots were visualized under a UV light detection (254 nm). Then TLC plates were sprayed with vanillin solution and heated at 150 °C for 5 min to detect octyl and cetyl esters.

Structures of the purified octyl and cetyl esters were confirmed by nuclear magnetic resonance (NMR) spectroscopy. ^1^H and ^13^C NMR spectra of the isolated esters were recorded at 700 MHz and 170 MHz, respectively on a Bruker Avance III 700 MHz spectrometer (Bruker Corporation, Karlsruhe, Germany) at 298 ± 1 K. The samples were dissolved in chloroform-d (CDCl_3_) containing tetramethylsilane (TMS) as internal standard. Chemical shifts were recorded in δ values in parts per million (ppm) and coupling constant (*J*) were reported in Hertz (Hz).

The synthesized phenolic acids esters had the following characteristics:

#### OSA (octyl (E)-3-(4-hydroxy-3,5-dimethoxyphenyl)propenoate)

Light yellow oil, ^1^H NMR (CDCl_3_): δ 0.88 (t, *J* = 7.0 Hz, 3H), 1.23–1.36 (m, 8H), 1.37–1.43 (m, 2H), 1.66–1.73 (m, 2H), 3.91 (s, 6H), 4.19 (t, *J* = 6.8 Hz, 2H), 5.83 (br. s., 1H), 6.30 (d, *J* = 15.9 Hz, 1H), 6.77 (s, 2H), 7.58 (d, *J* = 15.9 Hz, 1H). ^13^C NMR (CDCl_3_): δ 13.80, 22.38, 25.75, 28.52, 29.01, 31.55, 56.02 (2xOCH_3_), 64.38, 104.92 (2xCH), 115.58, 125.59, 137.11, 144.70, 147.11 (2 × C), 167.09.

#### OFA (octyl (E)-3-(4-hydroxy-3-methoxy-phenyl)-2-propenoate)

Light cream oil, ^1^H NMR (CDCl_3_): δ 0.89 (t, *J* = 7.1 Hz, 3H), 1.24–1.37 (m, 8H), 1.38–1.43 (m, 2H), 1.67–1.73 (m, 2H), 3.92 (s, 3H), 4.20 (t, *J* = 6.7 Hz, 2H), 5.98 (br. s., 1H), 6.30 (d, *J* = 15.9 Hz, 1H), 6.92 (d, *J* = 8.2 Hz, 1H), 7.03 (d, *J* = 1.9 Hz, 1H), 7.07 (dd, *J* = 8.2, 1.9 Hz, 1H), 7.61 (d, *J* = 15.9 Hz, 1H). ^13^C NMR (CDCl_3_): δ 14.00, 22.58, 25.94, 28.72, 29.13, 29.19, 31.73, 55.86, 64.57, 109.36, 114.73, 115.58, 122.95, 126.98, 144.62, 146.79, 147.93, 167.38.

#### OCA (octyl (E)-3-(3,4–dihydroxyphenyl)-2-propenoate)

White solid, ^1^H NMR (CDCl_3_): δ 0.90 (t, *J* = 7.2 Hz, 3H), 1.24–1.37 (m, 9H), 1.37–1.43 (m, 2H), 1.67–1.74 (m, 2H), 4.20 (t, *J* = 6.8 Hz, 2H), 5.59 (br. s., 2H), 6.28 (d, *J* = 15.9 Hz, 1H), 6.88 (d, *J* = 8.2 Hz, 1H), 7.03 (dd, *J* = 8.2, 1.9 Hz, 1H), 7.10 (d, *J* = 1.9 Hz, 1H), 7.58 (d, *J* = 15.9 Hz, 1H). ^13^C NMR (CDCl_3_): δ 14.06, 22.64, 25.98, 28.74, 29.19, 29.24, 31.79, 64.75, 114.40, 115.57, 116.12, 122.44, 127.88, 143.66, 144.39, 145.98, 167.56.

#### CSA (hexadecyl (E)-3-(4-hydroxy-3,5-dimethoxyphenyl)propenoate)

Light yellow solid, ^1^H NMR (CDCl_3_): δ 0.84 (t, *J* = 7.7 Hz, 3H), 1.22–1.31 (m, 25H), 1.36–1.37 (m, 2H), 1.66–1.68 (m, 2H), 3.88 (s, 6H), 4.16 (t, *J* = 7.0 Hz, 2H), 5.88 (br. s., 1H), 6.27 (d, *J* = 16.1 Hz, 1H), 6.74 (s, 2H), 7.55 (d, *J* = 16.1 Hz, 1H). ^13^C NMR (CDCl_3_): δ 14.06, 22.65, 28.74, 29.30–29.67 (12 CH_2_), 31.89, 56.15 (2x×OCH_3_), 64.54, 104.99 (2×CH), 115.74, 125.77, 137.21, 144.86, 147.24 (2×C), 167.20.

#### CFA (hexadecyl (E)-3-(4-hydroxy-3-methoxy-phenyl)-2-propenoate)

Light orange solid, ^1^H NMR (CDCl_3_): δ 0.88 (t, *J* = 7.7 Hz, 3H), 1.25–1.31 (m, 28H), 1.36–1.37 (m, 2H), 1.68–1.69 (m, 2H), 3.93 (s, 3H), 4.18 (t, *J* = 14 Hz), 5.86 br. s., 1H), 6.30 (d, *J* = 16.1 Hz, 1H), 6.91 (d, 8.4 Hz, 1H), 7.03 (d, 2.1 Hz, d), 7.06 (dd, *J* = 9.6, 2.1 Hz, 1H), 7.59 (d, *J* = 16.1 Hz, 1H). ^13^C NMR (CDCl_3_): δ 14.10, 22.69, 25.74, 26.00, 28.61, 28.79, 29.31, 29.36, 29.46, 29.55, 29.59, 29.65, 29.69, 31.92, 32.83, 55.94, 63.12, 64.61, 109.31, 114.68, 115.73, 123.09, 144.06, 144.76, 146.76, 147.90, 167.37.

### Samples

Refined rapeseed oil in the original package (1-L polyethylenterephthalate (PET) bottle) was kindly provided by a local vegetable oil factory. The frozen pre-fried French fries (Aviko brand, purchased from a local supermarket) were selected as the standard test food for frying due to their standard composition (an initial fat content = 4.0%, protein = 2.5%, carbohydrates = 24.5%) and easy availability.

### Frying procedure

Rapeseed oils enriched with OSA, OFA, OCA, CSA and CFA, respectively (100.0 g of oil, c_phenolipid_ = 0.5%) were poured into 250 mL glass beakers (6.0 cm diameter) and heated up to 180 ± 5ºC on a hot plate for 10 min. The oil temperature was checked by a digital thermometer. Then portion of 15.0 ± 0.5 g of frozen French fries (approximately 9 × 9 × 30 mm) was immersed into each oil and fried for 4 min. After frying under domestic conditions, French fries were placed in a clean dry paper for 5 min, allowing for the excess oil to drain. All frying experiments were performed in two repetitions using control and enriched rapeseed oils as well as frozen French fries. The fried oils in amber-colored bottles and fried French fries collected in aluminium foil were stored in refrigerator (4ºC) for 1 day until analysis.

### Determination of antioxidant activity and total phenolic content

The AA and TPC in methanolic extracts of fortified rapeseed oils used as frying media, fried French fries and oils extracted from them were determined by modified spectrophotometric methods: ABTS, DPPH, FRAP and Folin–Ciocalteu (FC), respectively described in our previous report (Szydłowska-Czerniak and Łaszewska 2015).

The detailed procedures of samples preparation and the AA and TPC determination can be found in the supplementary data.

### Oil content in French Fries

The oil from French fries was extracted three times by hexane using a Soxhlet apparatus. Briefly, the grounded French fries (5.0 g) were packed inside a cartridge, transferred to an extractor device and submitted to 3 h recycling extraction with 150 mL hexane at boiling temperature (70 °C). The hexane-oil mixtures were heated to evaporate the solvent and the oil was weighted using an analytical balance. Yield of oil was expressed as:

$$\% {\text{oil content } = \text{ }}\frac{{\text{weight of oil obtained after extraction}}}{{\text{weight of dry sample}}} \times 100$$

### Analysis of oils oxidative status

The refined and supplemented rapeseed oils before and after frying were characterized according to the official ISO methods by peroxide value (PV) (ISO 3960 2017), anisidine value (p-AnV) (ISO 6885 2016), acid value (AV) (ISO 660 2009), conjugated diene (CD) and conjugated triene (CT) values as the absorbance of 1% solution of each oil in hexane (no additional dilutions were made) at 233 and 268 nm, respectively in a 1 cm quartz cell (ISO 3656 2011). The TOTOX was used as a characteristic value for total oxidation of triglycerides and was calculated by the expression: TOTOX = (2PV + p-AnV).

### Statistical analysis

The AA and TPC in oils before and after frying, French fries and oils extracted from them were determined five times within 1 day by the modified ABTS, DPPH, FRAP and FC methods. The oil content in French fries as well as oxidation parameters: PV, p-AnV, AV and amounts of CD and CT in each oils before and after frying were analyzed in triplicate. The obtained results were presented as: mean (c) ± standard deviation (SD). One-way analysis of variance (ANOVA), followed by the Duncan test, was performed to analyze the significant differences between data (*p* < 0.05).

## Results and discussion

### Antioxidant activity and total phenolic content in frying oils and French Fries

The AA determined by three analytical methods was the highest for rapeseed oil with OCA and the lowest for control oil without antioxidants (Table 1).

**Table 1:** Antioxidant activity and total phenolic content in the refined (RO) and enriched rapeseed oils before (BF) and after (AF) frying process

Oil sample	ABTS* ± SD (µmol TE/100 g)	DPPH* ± SD (µmol TE/100 g)	FRAP* ± SD (µmol TE/100 g)	TPC* ± SD (mg SA/100 g)
BF-RO	905 ± 42^a^	322 ± 3^a^	296 ± 11^b^	12 ± 0.5^a^
AF-RO	806 ± 33^a^	313 ± 14^a^	227 ± 4^a^	10 ± 0.4^a^
BF-RO + OSA	13,850 ± 633^ g^	3794 ± 93^ h^	980 ± 21^i^	167 ± 4^ g^
AF-RO + OSA	12,328 ± 239^f^	3505 ± 115^ g^	817 ± 37^ h^	138 ± 5^f^
BF-RO + OFA	9406 ± 132^d^	2870 ± 96^e^	557 ± 6^f^	134 ± 3^f^
AF-RO + OFA	10,063 ± 281^e^	3209 ± 86^f^	646 ± 24^ g^	136 ± 5^f^
BF-RO + OCA	15,621 ± 591^ h^	7628 ± 247^j^	3327 ± 34^ k^	607 ± 17^i^
AF-RO + OCA	15,266 ± 538^ h^	6374 ± 107^i^	1658 ± 20^j^	351 ± 12^ h^
BF-RO + CSA	6434 ± 109^b,c^	1792 ± 22^c^	439 ± 21^e^	69 ± 3^d^
AF-RO + CSA	6584 ± 122^b,c^	2600 ± 118^d^	676 ± 20^ g^	86 ± 4^e^
BF-RO + CFA	6146 ± 170^b^	1493 ± 26^b^	340 ± 13^c^	34 ± 1^b^
AF-RO + CFA	6832 ± 222^c^	1842 ± 69^c^	375 ± 16^d^	45 ± 2^c^

*n = 5; *SD* standard deviation; Different letters (a–k) within the same column indicate significant differences between AA and TPC in oils

Furthermore, the AA results revealed that octyl esters had significantly higher antioxidant potential than cetyl esters. Therefore, the ABTS, DPPH and FRAP values of rapeseed oils enriched with octyl esters were more than two times higher than those for rapeseed oils fortified with cetyl esters (Table 1, Duncan test).

Nevertheless, the FRAP and DPPH of oil with phenolipids changed significantly during frying process, whereas ABTS of oils with OCA and CSA did not differ significantly before and after frying (Table 1, Duncan test). This indicated that the hydrophilic antioxidants determined by FRAP assay and these hydrophobic compounds available to react with the DPPH radical were not thermally stable and were reduced during frying, but the fried oils were all time in contact with the most sensitive components of French fries (vitamins, phenolics and other antioxidants), which can affected total antioxidant potential of oils. The supplementation of rapeseed oil with OSA resulted in a more effective protection of hydrophobic antioxidants against their decomposition (the lowest DPPH decrease was observed for oil with OSA after frying). However, OCA can be the most protective against both hydrophilic and hydrophobic antioxidants (the ABTS of oil with OCA insignificantly reduced after frying).

On the contrary, Duncan test indicated that the AA of oils fortified with OFA, CSA and CFA significantly increased after frying (Table 1). This fact can be explained as that both cetyl esters and OFA probably retarded degradation of antioxidants naturally present in rapeseed oil as well as bioactive compounds from French fries, which contact with fortified oils during frying. Moreover, cetyl esters could be less thermally stable under the drastic conditions than octyl esters and easily hydrolized to corresponding phenolic acids having a high antioxidant potential.

It can be noted that frying in oils without phenolipids and with OSA and OCA reduced TPC in the studied oils by about 17–42%, whereas oils enriched with CSA and CFA contained approximately 1.3 times higher amount of phenolics after processing (Table 1). It is known that thermal treatment of oil causes a decrease in phenolics content, but the presence of cetyl esters and endogenous phenolics in rapeseed oil and French fries can enhance their relative stability. The Duncan test indicated that, frying in rapeseed oil with OFA did not affect significantly TPC, which was similar to this found in the fried rapeseed oil with OSA. Also, TPC in control oil did not change significantly during frying.

For comparison, sunflower (IC_50_ = 13.2–65.3 mg), olive (IC_50_ = 12.5–35.1 mg) and palm (IC_50_ = 19.9–69.1 mg) oils enriched with olive leaf extract revealed higher DPPH values than control samples (IC_50_ = 91.3–98.9 mg, 30.9–40.7 mg and 75.2–139.4, respectively) before and after frying. Although the quantities of all fried oils needed to decrease the initial DPPH radical concentration to 50% were higher than for fresh oils (Chiou 2009). Furthermore, rapeseed oils supplemented with olive leaf, hazelnut leaf and hazelnut green leafy cover extracts had higher ABTS results (168.2, 109.9 and 199.8 μmol TE/g) at the end of frying than control sample (56.3–103.9 μmol TE/g) in the all frying days. However, TPC in rapeseed oils with olive leaf and hazelnut green leafy cover extracts ranged between 2 and 8 mg/100 g and 1–6 mg/100 g, respectively until the end of 7 days frying at 180 °C, while phenolics were lost in rapeseed oil enriched with hazelnut leaf extract. Although the same amounts of phenolics remained in oil with hazelnut green leafy cover extract after first and seventh day of frying (Aydeniz and Yilmaz 2012). The concentrations of total phenolics in several oils decreased after frying at 180 °C. However, lower differences in TPC were noted for soybean oil with rosemary (28%) than in control oil sample (39%) (Saoudi et al. 2016).

Rapeseed oils without and with five synthesized phenolipids were used to evaluate the effect of frying media on the AA and TPC in French fries. The obtained results of ABTS, DPPH, FRAP and TPC in French fries are listed in Table 2.

**Table 2:** Antioxidant activity and total phenolic content in French fries fried in different media: refined (RO) and enriched rapeseed oils

Frying medium	ABTS* ± SD (µmol TE/100 g)	DPPH* ± SD (µmol TE/100 g)	FRAP* ± SD (µmol TE/100 g)	TPC* ± SD (mg SA/100 g)
RO	2146 ± 28^a^	403 ± 6^a^	218 ± 8^a^	14 ± 0.6^a^
RO + OSA	4992 ± 176^c^	980 ± 18^c,d^	612 ± 26^d^	172 ± 3^e^
RO + OFA	2907 ± 99^b^	903 ± 16^c^	385 ± 19^b^	96 ± 0.5^c^
RO + OCA	20,029 ± 871^d^	6886 ± 205^e^	2659 ± 123^e^	378 ± 4^f^
RO + CSA	3409 ± 169^b^	1046 ± 33^d^	527 ± 19^c^	141 ± 5^d^
RO + CFA	3263 ± 49^b^	657 ± 29^b^	332 ± 4^b^	44 ± 1^b^

*n = 5; *SD* standard deviation; Different letters (a–f) within the same column indicate significant differences between AA and TPC in French fries fried in different media

The AA of fried French fries ranged between 2146 and 20,029 μmol TE/100 g, 403–6886 μmol TE/100 g and 218–2659 μmol TE/100 g for the ABTS, DPPH and FRAP assays, respectively. Nevertheless, TPC determined by the FC method was the lowest for French fries prepared in refined rapeseed oil (14 mg SA/100 g) and the highest for fries fried in rapeseed oil fortified with OCA (378 mg SA/100 g). As expected, higher antioxidant potential of oil used for frying increased the AA and TPC in French fries (Tables 1 and 2). French fries fried in the supplemented oils revealed approximately 2–27 times higher AA and TPC results than those prepared in the refined rapeseed oil. Therefore, the frying medium significantly affected the AA and TPC in French fries (Table 2, Duncan test). This can be explained by the retention of antioxidants by French fries processed in oils enriched with phenolipids. The AA of French fries is composite mixture of all antioxidants from fries together with those from frying oil, because a part of oil is absorbed by fries during frying. However, antioxidants degraded under heating conditions, thus the AA of French fries is not sum of antioxidants found in oil and fries. Insignificant differences in ABTS and FRAP results were observed between French fries fried in rapeseed oils with ferulic acid esters, whereas the DPPH of fries prepared in oils containing CSA, OSA and OFA did not differ significantly (Table 2, Duncan test).

Similar effect of olive leaf extract added to sunflower, olive and palm oils on the DPPH and TPC in French fries was demonstrated by Chiou et al. (2007, 2009). French fries fried in supplemented sunflower, olive and palm oils had higher AA (EC_50_ = 19.9–37.7, 15.2–32.0 and 31.5–174 mg) and TPC (19.3–23.2, 13.8–15.2 and 9.1–11.1 mg/100 g, respectively) than the respective ones prepared in non-supplemented oils (EC_50_ = 57.2, 54.0 and 454 mg, while TPC = 7.8, 9.4 and 8.2 mg/100 g, respectively).

The analytical procedures for determinations of AA and TPC in French fries and oils before and after frying process do not significantly differ in their precisions (the relative standard deviation (RSD) results varied between 1.30–4.96%, 0.93–4.54%, 1.02–4.94%, and 0.52–4.65% for ABTS, DPPH, FRAP and FC methods respectively), thus the proposed methods can be applied for reliable assessment of total antioxidant potential and phenolics content in the studied samples.

## Quantity and antioxidant activity of oil absorbed by French Fries

**Fig. 1:**
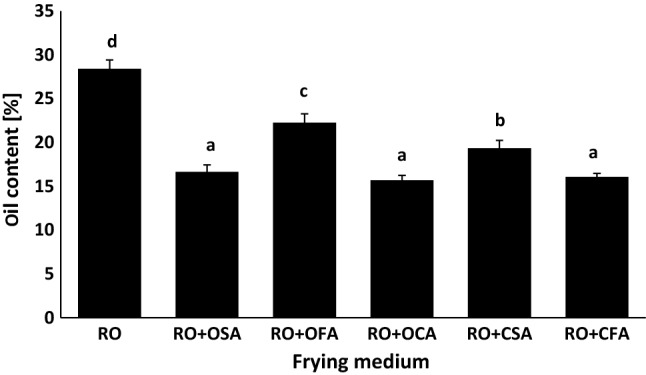
Oil absorption during frying of French fries in different frying media: refined (RO) and enriched rapeseed oils. Different letters in the superscripts mean significant differences between used frying medium

The amount of oil absorbed by fried products is a critical parameter of their quality. As seen, the type of frying medium had a significant effect on the evolution of oil uptake in French fries (Fig. 1, Duncan test).

High fat content in the fried French fries (15.7–28.4%) can be explained by the fact that frozen French fries were used for frying. Freezing increased the fat absorption due to lower amount of water in the frozen samples. Moreover, higher oil content in frozen samples after frying indicates that freezing temperature damaged their structures and enhanced fat absorption (Adedeji and Ngadi 2018). Oil content was the highest in French fries processed in the refined rapeseed oil without phenolipids (28.4%). Oil uptake was importantly reduced (22–45%) when phenolipids were added to oil. It is noteworthy that the lowest and similar oil absorption revealed French fries prepared in oils supplemented with OCA, OSA and CFA (Fig. 1). Probably, these phenolipids were more thermally stable and effectively reduced the dehydration of French fries. The hydrating effect of antioxidants added to frying medium facilitated starch gelatinization, improving structure formation and decreasing oil uptake by French fries.

Interestingly, the ABTS, DPPH, FRAP and TPC results of oils extracted from French fries after frying in rapeseed oil without phenolipids, with CSA and CFA were about 1.3–3.3 times higher than AA and TPC in these three oils used for frying (Tables 1 and 3).

**Table 3:** Antioxidant activity and total phenolic content in oils extracted from French fries fried in different media: refined (RO) and enriched rapeseed oils

Frying medium	ABTS* ± SD (µmol TE/100 g)	DPPH* ± SD (µmol TE/100 g)	FRAP* ± SD (µmol TE/100 g)	TPC* ± SD (mg SA/100 g)
RO	2304 ± 96^a^	443 ± 14^a^	386 ± 15^a^	20 ± 0.7^a^
RO + OSA	8946 ± 284^d^	3354 ± 44^e^	1234 ± 20^c^	145 ± 7^d^
RO + OFA	2857 ± 50^b^	697 ± 28^b^	402 ± 5^a^	42 ± 1^b^
RO + OCA	6050 ± 206^c^	2491 ± 96^c^	1148 ± 52^b^	134 ± 6^c^
RO + CSA	9447 ± 446^e^	3425 ± 89^e^	1329 ± 21^d^	143 ± 1^d^
RO + CFA	8633 ± 211^d^	3137 ± 136^d^	1232 ± 16^c^	131 ± 5^c^

*n = 5; *SD* standard deviation; Different letters (a–e) within the same column indicate significant differences between AA and TPC in oils from French fries fried in different media

However, oils absorbed by French fries had significantly lower AA (4–78%) and TPC (about 60%) than fried oils with octyl esters. This suggests that cetyl esters had a higher ability for dissolving of antioxidants present in French fries and supplemented oils when compared to octyl esters. Probably, the addition of cetyl esters enhanced the retention of antioxidant compounds in oils and their diffusion rate from fries to the extracted oil. On the other hand, oil extracted from French fries fried in refined rapeseed oil revealed significantly lower values of AA and TPC then those obtained from fries prepared in the fortified rapeseed oils (Table 3, Duncan test). This can be explained by the fact that phenolipids introduced to oil were capable to protect the bioactive compounds present in French fries and improve their retention.

In addition, the Duncan test indicated that the extracted oils from fries processed in rapeseed oils with OSA and CFA did not differ significantly in the ABTS and FRAP results. Insignificant differences in the DPPH and TPC were found between oils absorbed after frying in rapeseed oils with SA esters. Also, there were no significant differences in FRAP for oils extracted from fries fried in rapeseed oils without phenolipids and with OFA, as well as values of TPC for oils absorbed after frying in oils with OCA and CFA (Table 3, Duncan test).

Although there is a lack of information in the literature about the changes in the AA and TPC in oils extracted from food products after frying, some authors estimated the effects of olive leaf, hazelnut leaf and hazelnut green leafy cover added to sunflower, olive, palm and canola oils on oil content in the fried French fries and dough (Aydeniz and Yilmaz 2012; Chiou et al. 2007, 2009). Addition of phenolics from olive leaf to sunflower and olive oils reduced the oil amount in French fries by 3–16%, whereas French fries after frying in enriched palm oil had higher fat content (14.2–15.5 g/100 g) than those prepared in palm oil without phenolics (11.6 g/100 g) (Chiou et al. 2007, 2009). Moreover, the oil uptake in dough after the first three days frying in canola oils fortified with olive and hazelnut leaf extracts was higher than in refined canola oil, while dough fried the fourth and fifth day contained lower fat (Aydeniz and Yilmaz 2012).

## Oxidation state of frying oils

It is known that the consumption of products from oxidized oils poses a chronic threat to human health. For this reason, the oxidation state of rapeseed oils with novel antioxidants after frying of French fries was estimated by analyzing amounts of primary (PV, CD, CT) and secondary (p-AnV) oxidation products, free fatty acids content (AV) and total oxidation (TOTOX) index (Table 4).

**Table 4:** Oxidation parameters of the refined (RO) and enriched rapeseed oils before (BF) and after (AF) frying process

Oil sample	PV* ± SD (meq O_2_/kg)	p-AnV* ± SD	TOTOX	CD* ± SD	CT* ± SD	AV* ± SD (mg NaOH/g)
BF-RO	0.08 ± 0.002^a^	1.51 ± 0.07^a^	1.67^a^	2.606 ± 0.125^a^	0.489 ± 0.018^a^	0.015 ± 0.0003^a^
AF-RO	11.24 ± 0.11^ h^	8.74 ± 0.26^e^	31.22^ h^	2.837 ± 0.048^b^	0.672 ± 0.030^b^	0.049 ± 0.002^b^
BF-RO + OSA	1.54 ± 0.06^c^	1.63 ± 0.05^a^	4.71^c^	3.001 ± 0.043^c^	0.930 ± 0.043^d^	0.130 ± 0.004^f^
AF-RO + OSA	6.37 ± 0.04^f^	7.28 ± 0.29^b^	20.02^f^	3.200 ± 0.034^e,f^	1.139 ± 0.008^f^	0.250 ± 0.005^j^
BF-RO + OFA	0.42 ± 0.01^b^	1.64 ± 0.06^a^	2.48^b^	3.121 ± 0.044^d,e^	1.007 ± 0.038^e^	0.110 ± 0.004^e^
AF-RO + OFA	3.73 ± 0.10^d^	7.91 ± 0.11^c^	15.37^d^	3.282 ± 0.042^f^	1.135 ± 0.005^f^	0.260 ± 0.002^ k^
BF-RO + OCA	0.19 ± 0.009^a^	1.54 ± 0.01^a^	1.92^a^	3.087 ± 0.010^c,d^	1.573 ± 0.052^i^	0.150 ± 0.003^ g^
AF-RO + OCA	7.69 ± 0.21^ g^	8.41 ± 0.25^d^	23.79^ g^	3.229 ± 0.048^f^	2.037 ± 0.015^j^	0.240 ± 0.004^i^
BF-RO + CSA	0.12 ± 0.003^a^	1.78 ± 0.05^a^	2.02^a^	3.070 ± 0.006^c,d^	0.857 ± 0.032^c^	0.060 ± 0.001^c^
AF-RO + CSA	4.85 ± 0.07^e^	7.81 ± 0.20^c^	17.51^e^	3.246 ± 0.014^f^	0.979 ± 0.016^e^	0.130 ± 0.006^f^
BF-RO + CFA	1.59 ± 0.04^c^	1.73 ± 0.05^a^	4.91^c^	3.081 ± 0.017^c,d^	1.365 ± 0.011^ g^	0.080 ± 0.002^d^
AF-RO + CFA	4.90 ± 0.07^e^	8.07 ± 0.13^c^	17.87^e^	3.212 ± 0.036^f^	1.457 ± 0.034^ h^	0.170 ± 0.005^ h^

*n = 3; *SD* standard deviation; Different letters (a–k) within the same column indicate significant differences between oxidation parameters of oils

It is noteworthy that the unheated rapeseed oil revealed the lowest PV = 0.08 meq O_2_/kg and p-AnV = 1.51, while the addition of phenolipids caused an increase in amounts of hydroperoxides (PV = 0.12–1.59 meq O_2_/kg) and compounds with aldehyde carbonyl bond (p-AnV = 1.54–1.78) in rapeseed oil. The PV and p-AnV of oils before frying were below the maximum values (PV < 5 meq O_2_/kg and p-AnV < 8) permitted for vegetable oils according to the ISO 3960 (2017) and ISO 6885 (2016). However, the heated rapeseed oils without esters and with OSA and OCA presented PV above the legal limit (6.37–11.24 meq O_2_/kg). The use of novel antioxidants significantly decreased the PV of oils after frying (PV = 3.73–7.69 meq O_2_/kg) when compared to the control oil (PV = 11.24 meq O_2_/kg). Moreover, p-AnV of all fried rapeseed oils were approximately 5 times higher than for oils before frying (Table 4). A slower increase of PV and p-AnV for the fortified rapeseed oils compared to a non-fortified oil indicates the effectiveness of novel antioxidants in delaying the oil oxidation. The changes in PV during frying of French fries depended on type of phenolipid added to oil, however oils with CFA and OSA oxidized at a slower rate. The PV of refined rapeseed oil and after addition of OCA and CSA were not significantly changed (Table 4, Duncan test). Furthermore, similar content of primary oxidation products was determined in oils fortified with OSA and CFA as well as in the fried oils with cetyl esters. As seen, the addition of each phenolipid to rapeseed oil did not significantly change the content of secondary oxidation products in the enriched oils (Table 4, Duncan test). Insignificant differences in p-AnV were also observed between oils with OFA, CSA and CFA after frying.

Additionally, the significant increase in total oxidation (high values of TOTOX) was found in all oils during frying. The control rapeseed oil and oil with OCA were more oxidized than oils stabilized with other phenolipids (Table 4). This can be explained by lower amount of natural antioxidants in control sample, causing its high oxidation state, whereas OCA was less thermally stable compared to the other esters. In addition, the Duncan test indicated that there were significant differences between the effectiveness of the added octyl esters in retarding the deterioration process of oils during frying (Table 4).

On the other hand, CD and CT values, which represent the degree of production of the primary oxidation products in control rapeseed oil differed significantly from those determined for oils with phenolipids (Table 4, Duncan test). An increase in CD and CT amounts for control oil (8.1 and 27.2%, respectively) at the end of frying was higher than that for oils with synthesized antioxidants (4.1–6.2% and 6.3–22.8% for CD and CT, respectively). Although the supplementation with five different esters did not significantly affect the CD values of unheated and heated oils (Table 4, Duncan test).

As seen, the thermal oxidation and hydrolysis reactions induced by water introduced with French fries caused the decomposition of triglycerides into free fatty acids (FFA), resulting in the significant AV increase during the frying process (Table 4, Duncan test). All the studied oils before and after frying had AV (0.015–0.260 mg NaOH/g) below desirable level (AV < 0.300 mg NaOH/g) for refined oil. Unexpectedly, addition of phenolipids increased the AV of fortified oils, suggesting that these esters probably promoted the hydrolysis reactions and FFA production.

Also Matthäus et al. (2014) found higher concentration of FFA (0.36–1.00 g/100 g) in rapeseed oils supplemented with rosemary extracts and canolol than AV (0.10–0.60 g/100 g) of the fried rapeseed oil without antioxidants.

Although oils with cetyl esters had two times lower AV when compared to the AV of oils with octyl esters. However, an increase in AV during frying was significantly reduced (from 70% to 38–58%) after addition of novel antioxidants to oil. Hence, phenolipids can be used as additives for increasing the stability of the fried oils.

It is noteworthy that, the addition of phenolipids unexpectedly induced primary and secondary oxidation products formation in fresh rapeseed oil. Probably, intensive mixing of oils with antioxidants during enrichment caused an increase in PV, p-AnV, AV and level of conjugated polyenes. Moreover, the synthesized cinnamate derivatives strongly absorbed in the UVA (400–315 nm) and UVB (315–280 nm) regions of the electromagnetic spectra. Therefore, the official spectrophotometric methods with low-cost, more available instrument, high precision (RSD = 0.65–4.64, 0.20–4.80 and 0.44–4.62% for p-AnV, CD and CT, respectively, Table 4), but lower sensitivity due to overlapping peaks characteristic for added antioxidants and oxidation products were used for simple monitoring of oxidation products in the fortified oils. Absorbance at 268 nm provides a measure of the CT content, however the band at 270 nm for caffeates and ferulates corresponds to a mixture of n → π* and π → π* transitions (it consists of a charge transfer from the carboxylate and the carbonaceous chain toward the benzene ring) (Horbury et al. 2017).

It seems that CFA was the most efficient in the inhibition of generation of primary oxidation products in oil during frying, whereas OCA can be considered as more effective than others esters for FFA reduction in the heated rapeseed oil. However, the highest inhibitory effect on the formation of secondary oxidation products during frying determined as p-AnV was observed in the CSA and OSA (Table 4).

For comparison, rapeseed oils enriched with different natural (native phloridzin from Canadian crabapple, extracts from Rosa woodsii hip, Sorbus aucuparia and Malus baccata berries, rosemary, canolol as well as canola oil deodistillates) and synthetic (phloridzyl octadecanoate, dihydrocaffeic acid amides, BHT and TBHQ) antioxidants revealed significantly lower PV = 110–279 μM, p-AnV = 25–201, TOTOX = 100–994, CD = 1.00–4.19 and CT = 0.22–1.14 after frying process than oils without antioxidants (PV = 120–831 μM, p-AnV = 100–224, TOTOX = 390–1778, CD = 1.50–4.64 and CT = 0.39–3.18) (Aachary et al. 2014; Aladedunye et al. 2014; Aladedunye and Matthäus 2014, 2016; Aladedunye and Przybylski 2014; Matthäus et al. 2014; Redondo-Cuevas et al. 2017).

## Conclusion

The presence of novel antioxidants in rapeseed oil affected the antioxidant potential of fried French fries and oil uptake. The enrichment of rapeseed oil with phenolipids—octyl and cetyl esters of phenolic acids enhanced the AA and TPC in French fries. French fries during frying in the fortified oils absorbed lower amount of oil with higher AA and TPC.

Generally cetyl esters strongly inhibited the formation of oxidation products in rapeseed oil during frying under domestic conditions. However, oils fortified with octyl esters had higher antioxidant potential before and after frying process than oils with cetyl esters. Therefore, future activities should mainly focus on investigations of changes in the quality of fried products and oils enriched with phenolipds during continuous frying, which is industrially important food process.

Moreover, it is crucial to evaluate potential toxic effect of phenolipids added to oil-based foods and identify the impact of different processes such as frying and storage under various conditions that can be responsible for their toxicity.

## Electronic supplementary material

Below is the link to the electronic supplementary material.

Supplementary file1 (DOCX 17 kb)
