# Supplementary material for: Effect of new antioxidants: phenolipids on quality of fried French fries and rapeseed oil
Source: J Food Sci Technol. 2020 Sep 1;58(7):2589–98. doi: 10.1007/s13197-020-04765-z (PMC8196133; doi:10.1007/s13197-020-04765-z)
Supplement: Supplementary file 1 — Supplementary file1 (DOCX 17 kb) [file 13197_2020_4765_MOESM1_ESM.docx]

**Appendix A. Supplementary data**

**Samples preparation for determination of antioxidant activity and total phenolic content**

The test tubes with homogenized French fries (1.0 g) or oils (2.0 g) and methanol (5 mL and 10 mL, respectively) were shaken for 30 min at room temperature using a shaker SHKA 25081CE (Labo Plus, Warsaw, Poland). The extracts were separated from samples (in a refrigerator about 4°C for French fries and in a freezer below −20 °C for oils) and transferred quantitatively into glass bottles. Each sample was extracted in triplicate and methanolic extracts were stored in a refrigerator, prior to antioxidant activity (AA) and total phenolic content (TPC) analysis.

**Analytical methods for antioxidant activity and total phenolic content determination**

*ABTS method*

The ABTS^•+^ solution (7 mmol/L ABTS solution with 2.45 mmol/L potassium persulfate (1:0.5) kept for 16 h) was diluted with ethanol to an absorbance of 0.70 at 734 nm. Then, 0.01 – 0.14 mL of oil extracts or 0.06 – 0.5 mL of methanolic extract of French fries were filled up to 2.5 mL with ABTS^•+^ solution and mixtures were incubated at 30ºC for 5 min. The absorbance was measured at 734 nm against a reagent blank (2.5 mL of ABTS^•+^ solution).

*DPPH method*

Briefly, 0.01 – 0.2 mL of oil extracts or 0.1 – 1.0 mL of methanolic extract of French fries were filled up to 2.0 mL with methanol and added 0.5 mL of DPPH methanolic solution (304 µmol/L). The mixtures were shaken and left in darkness for 15 min. The absorbance was measured at 517 nm against a reagent blank (2.0 mL of methanol + 0.5 mL of DPPH methanolic solution).

*FRAP method*

The freshly prepared FRAP reagent (10 mmol/L 2,4,6-tris(2-pyridyl)-s-triazine solution (2.5 mL) in 40 mmol/L HCl + 20 mmol/L FeCl_3_ (2.5 mL) + 0.1 mol/L acetate buffer (25 mL), pH 3.6) was incubated at 40ºC for 15 min. Then, 0.01 – 0.17 mL of methanolic extracts of oils or 0.025 – 1.0 mL of methanolic extracts of French fries and 2.0 mL of the FRAP reagent were transferred into 10 mL volumetric flasks and made up to the mark with redistilled water. The obtained solutions were kept at dark place for 20 minutes. The absorbance was measured at 593 nm against a reagent blank (2.0 mL of the FRAP reagent made up to 10 mL with redistilled water).

*Folin-Ciocalteu method*

In brief, 0.75 – 1.30 mL of methanolic oil extracts or 0.60 – 1.75 mL of methanolic extracts of French fries and 1.00 mL of the FC reagent were transferred into 10 mL volumetric flasks, shaken for 3 min and 1.00 mL of saturated sodium carbonate solution (about 22%) was added and made up to the mark with redistilled water. After 1 h, solutions were centrifuged at 3120 × g (centrifuge MPW-54, MPW MED. INSTRUMENTS, Warsaw, Poland) for 15 min and absorbance at 765 nm was measured against a reagent blank (1.00 mL of FC reagent + 1.00 mL of saturated solution of Na_2_CO_3_ made up to 10 mL with redistilled water).

The UV–Vis spectra were recorded using a Hitachi U-2900 spectrophotometer (Tokyo, Japan) in a 1 cm quartz cell. The AA was expressed as μmol of Trolox equivalents per 100 g of sample, while TPC was given in mg of sinapic acid (SA) equivalents per 100 g of sample.
